# Herpes Simplex Type 1 Encephalitis Restricted to the Brainstem in a Pediatric Patient

**DOI:** 10.1155/2010/606584

**Published:** 2010-06-30

**Authors:** Juliana Harumi Arita, Jaime Lin, Mirella Maccarini Peruchi, Marcelo Masruha Rodrigues, Luiz Celso Pereira Vilanova

**Affiliations:** ^1^Division of Child Neurology, Department of Neurology and Neurosurgery, Universidade Federal de São Paulo, Rua Botucatu 720, 04023-900, São Paulo, Brazil; ^2^Department of Radiology, Faculdade de Medicina, Universidade São Paulo, Av. Dr. Arnaldo 455, 01246903, São Paulo, Brazil

## Abstract

Herpes simplex encephalitis is a potentially fatal infection of central nervous system that typically involves frontal and temporal lobes. Occasionally, it presents an extratemporal involvement and in rarer cases, it is limited to the brainstem. We describe a case of an adolescent who presented with fever, sore throat, and vertigo. Clinical picture evolved to lethargy, tetraparesis, consciousness impairment, and respiratory failure. MRI showed lesions restricted to the brainstem. PCR of CSF was positive for herpes simplex type 1.

## 1. Introduction

Herpes virus encephalitis (HSE) is one of the most severe infections of central nervous system. It is estimated to occur in 1/250000 to 1/500000 people per year, with children and adolescents accounting for one third of all cases [1–5]. 

Herpes simplex virus (HSV) is an ubiquitous human pathogen which can equally affect both sexes at any age [1]. HSV-1 infection typically starts in the limbic cortices and may then spread to adjacent frontal and temporal lobes resulting in acute inflammation, congestion, and/or hemorrhage. Less frequently, cingulated gyrus, basal ganglia, and parietal and occipital cortices are affected [1, 2, 6]. In very rare occasions, infection is limited to the brainstem and in these cases, cranial nerves abnormalities are the main manifestations [1, 7].

The identification of HSV by polymerase chain reaction (PCR) to detect virus DNA in cerebrospinal fluid (CSF) has become the diagnostic test of choice with a high sensitivity and specificity [1, 4–7]. However, in rare cases, false-negative results can occur [1, 5].

We report a case of HSV-1 encephalitis, in which CNS lesions evident on MRI were restricted to the brainstem.

## 2. Case Report

A 14-year-old male patient presented fever and sore throat five days prior to admission, followed by vertigo, nausea, and vomiting three days later. He was previously healthy, and there was no history of drug intake. On admission, he presented hyperemia in oropharynx and vesicles on upper lip. On neurological examination, lethargy, gait unsteadiness, nystagmus, and absence of vomiting reflex were observed. Therapy with 30 mg/kg/day of acyclovir and 4 g/day of ceftriaxone was performed. Despite the treatment, this patient evolved to tetraparesis, pyramidal signs, and consciousness impairment. Two days after admission, he went into respiratory failure and coma and required tracheal intubation. 

Conventional MRI was performed on a Magnetom Sonata Maestro Class 1,5 T scanner from Siemens using a parallel imaging head coil. The imaging protocol consisted of T1-weighted (TR, 453; TE, 13), T2-weighted (TR, 5219; effective TE, 100), fluid-attenuated inversion recovery (FLAIR) (TR, 8000; effective TE, 150), diffusion-weighted MR imaging (DWI; TR, 3513; effective TE, 70), and apparent diffusion coefficient (ADC) maps (TR, 3513; effective TE, 70).

**Figure 1:**
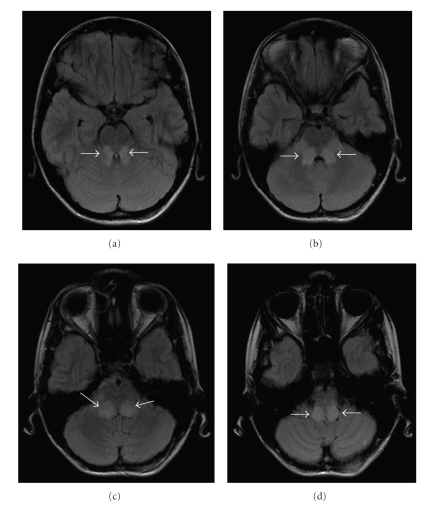
Axial fluid attenuation recovery (FLAIR) MR images show symmetric abnormal hyperintensity in the cerebellar tonsils and inferior, middle, and superior cerebellar peduncles.

**Figure 2:**
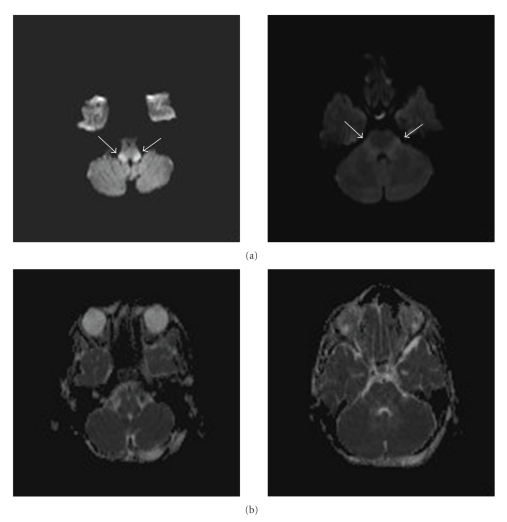
(a) Axial diffusion-weighted MR images show marked hyperintensity in some areas of the inferior and middle cerebellar peduncles. (b) On the apparent diffusion coefficient (ADC) maps, they are distinctly hypointense.

FLAIR MR images showed symmetric abnormal hyperintensity in inferior, middle, and superior cerebellar peduncles (Figure 1). Axial diffusion-weighted MR images showed marked hyperintensity in some areas of the inferior and middle cerebellar peduncles. On the apparent diffusion coefficient (ADC) maps, these areas were distinctly hypointense (Figure 2). Remaining cranial structures were spared with no other evident abnormalities.

CSF analysis showed 42 cells/mm_3_ with 89% lymphocytes, 8% monocytes, and 3% neutrophiles; glucose 86 mg/dL and protein 57 mg/dL. PCR amplification in CSF was positive for HSV-1 DNA. Investigation for immunodeficiencies was performed with negative results. 

The patient presented many episodes of apnea and bradypnea and was successfully extubated 1 month later. Acyclovir was administered for 21 days, and rehabilitation with physiotherapy and speech therapy was provided during hospitalization. After 45 days, he was discharged with mild dysarthria and nystagmus.

The hospital ethic committee approved this case report, and parents gave informed consent for publication.

## 3. Discussion

In this paper we describe a rare presentation of HSV-1 encephalitis in an immunocompetent pediatric patient. In the literature, there are few reports of this kind of infection with exclusive involvement of the brainstem. 

Extratemporal involvement in HSV encephalitis is not rare. In a recent study, 88 patients with HSV encephalitis were evaluated, and extratemporal lesions were present in 55% of them. However, it is interesting to note that in only 3 cases there was not an associated involvement of temporal lobes [8]. 

Miura et al. published a case with very similar image in a 53-year-old man probably secondary to a reactivation of HSV infection in the region of trigeminal nerve, not confirmed with positive HSV PCR [9]. As in our case, DW and ADC were performed revealing symmetrical lesions limited to the brainstem. Studies have demonstrated that these sequences may be more sensitive in the detection of HSV involvement than conventional MRI [9–12].

The gold standard to confirm HSV encephalitis is the detection of HSV DNA by PCR with an estimated sensitivity of 96% and specificity of 99% for early diagnosis in the general population. However, some studies have shown an increasing number of case reports with false negative PCR results, particularly in children, in which HSV PCR sensitivity can reach only 70%–75%. This fact could be related to the absence of HSV or the presence of a very low viral load in the CSF at the onset of an acute encephalitic process in children, maybe due to the presence of heme and other inhibitors [6]. 

If given early in the clinical course of HSE, acyclovir reduces both mortality and morbidity in treated patients. It should be administered at a dosage of 10 mg/kg every 8 hours (30 mg/kg/d) for a period of 14 to 21 days [2, 13].

Radiologically, it is important to remind as differential diagnosis postinfectious brainstem encephalitis, demyelinating disorders, brain tumors, infarcts, and metabolic diseases, such as mitochondriopathies [14]. In this paper, clinical presentation, MRI with DW and ADC sequences, as much as the positive result for HSV DNA by PCR in a pleocytic cerebrospinal fluid led us to the diagnosis of this rare occurrence.
